# Knowledge, attitude, and practice of medication use among pregnant women in Riyadh City: a cross-sectional study

**DOI:** 10.3389/fgwh.2024.1402608

**Published:** 2024-07-24

**Authors:** Alanoud Almuhareb, Abdullah Al Sharif, Peter Cahusac

**Affiliations:** College of Medicine, Alfaisal University, Riyadh, Saudi Arabia

**Keywords:** pregnancy, awareness, medication use, public health, Saudi Arabia

## Abstract

**Introduction:**

Medication use during pregnancy is limited by the scarcity of safety data for many drugs. The use of certain drugs during pregnancy can be teratogenic. Overestimating teratogenic medication risk could have serious consequences from drug non-adherence. Assessing and understanding the knowledge, attitude, and practice of medication use among pregnant women is crucial to optimizing the health of pregnant women and their fetuses.

**Methodology:**

An observational cross-sectional study used convenience and snowball sampling with a self-administered online questionnaire in 562 pregnant women from Riyadh City. The questionnaire used was adapted from previously published surveys. The survey included sections on sociodemographic background, awareness of medication risks, medication use during pregnancy, sources of drug information, and statements from the Beliefs about Medicines Questionnaire (BMQ), both general and pregnancy-specific.

**Results:**

Medication use during pregnancy was reported by 44.7%. The primary source for medication information for the majority was the physician. Over 58% indicated inadequate or inconsistent information about medication from physicians. Additionally, 65.7% confirmed not receiving sufficient or inconsistent information from pharmacists during medication dispensing. The responses to the questionnaire reveal a commendable level of knowledge and positive attitude and practice. However, variations could be found in these responses. Overall, no evident relationships were observed between predictors and responses, except in specific statements that indicated a positive association between beliefs and higher levels of education and youth.

**Conclusion:**

The results suggest a positive knowledge, attitude, and practice level. However, there was hesitancy and a restrictive attitude towards medication during pregnancy. The study identified inadequate education provided by healthcare professionals, thus presenting an area for improvement to enhance the safety and efficacy of medication use during pregnancy.

## Introduction

Pregnancy is a distinctive physiological state that necessitates meticulous consideration regarding medication use. The altered pharmacokinetics during pregnancy create challenges and concerns when determining the safety of medications for both the mother and the developing fetus. Nonetheless, the use of medications is often essential to treat acute medical conditions during pregnancy (1, 2). Over the past four decades, there has been a noticeable increase in medication use among pregnant women, whether prescribed or non-prescribed. This trend can be attributed to the fact that many women are now becoming pregnant at an older age, often with pre-existing chronic medical conditions that require ongoing treatment (1, 3). In one study, it was found that four out of five pregnant women were prescribed one or more medications, ranging from multivitamins and supplements to over-the-counter drugs and various prescribed medications to treat different medical conditions (4). Another study conducted among pregnant women at an ambulatory care clinic revealed that 39.3% of participants had at least one chronic medical condition necessitating drug therapy (5).

However, the use of medication during pregnancy is constrained by the lack of comprehensive safety data, complicating clinical decisions and posing health risks to both the mother and the fetus. This scarcity of data stems from the thalidomide tragedy in the early 1960s, where the drug, used as an antiemetic for pregnant women, led to a high incidence of phocomelia—congenital limb malformations occurring in 20%–30% of cases (6). The teratogenic effects of thalidomide prompted legal and regulatory decisions to exclude pregnant women from clinical trials, adversely affecting drug development and resulting in unreliable data with added safety concerns related to drug teratogenicity (7).

Teratogenic substances are defined as substances that can negatively affect embryo or fetal development if administered to pregnant women. These substances can harm the embryo or fetus in several ways, causing congenital malformations, behavioral or emotional development issues, and reduced intellectual quotient in the child. Additionally, teratogens can also lead to pregnancy complications, such as preterm labor, or abortions (8, 9). Major congenital malformations are reported in 3% of all newborns, with only 2%–4% of these anomalies attributed to drugs (9). Numerous substances, including certain medications, have been identified as teratogens (10).

Medication beliefs significantly influence medication adherence, reflecting compliance with prescriber instructions. Instruments like the Beliefs About Medicines Questionnaire (BMQ) have been developed to assess these beliefs (11). Several studies have documented the overestimation of teratogenic risks associated with medications (12, 13). A large-scale multinational survey encompassing 18 countries from Europe, North America, and Australia found that pregnant women often overestimate the risks of antidepressants and antibiotics (14). Such overestimations can lead to medication non-adherence, which is particularly critical in managing chronic diseases. Non-adherence can result in severe consequences for both the pregnant woman and the fetus, including fetal death and pregnancy termination (15).

Medication non-adherence in pregnant women is a well-documented issue, with various studies highlighting that the adherence rate among pregnant women with chronic diseases is approximately 40%. This low adherence rate leads to suboptimal management of chronic health conditions. Women generally perceive medication use during pregnancy as potentially harmful to the fetus, which makes them reluctant to use prescribed drugs during this period. Several factors contribute to this behavior, including personal beliefs and health literacy (16). One study investigating why pregnant women choose not to treat nausea and vomiting with pharmacologic medications found that concerns about potential teratogenic effects lead many to opt for non-pharmacologic alternatives, such as herbal remedies, which are perceived to carry fewer safety risks (17). In one study by Alkhaldi and Alkhammash involving pregnant women from Taif City, Saudi Arabia, the prevalence of using herbal medicines during pregnancy was 32.9%. Around 65% of these women believed that herbal medicines were unsafe for them and their unborn babies, compared to 75% for pharmacological therapies during pregnancy (18). These behaviors and attitudes toward medication use during pregnancy have garnered attention and have been studied in multiple countries.

Healthcare professionals need to understand the restrictive attitudes and practices of pregnant women regarding medication use to provide optimal and safe patient care. For instance, in developing countries, it has been reported that pregnant women frequently self-medicate without a prescription due to a lack of knowledge and awareness (19).

Given these circumstances, pregnant women often seek information from various sources with varying degrees of accuracy and reliability (20). Studies have shown that pregnant women frequently rely on online sources for information about pregnancy and medications, reflecting a growing desire to access accurate and relevant information conveniently (21, 22). One review revealed that pregnant women often view online health information as reliable and helpful for making informed decisions about their well-being (23). However, caution is necessary, as inappropriate and inconsistent information regarding the safety of medications during pregnancy is common on many websites. Encountering conflicting online information can lead to medication reluctance and increased anxiety (20). This issue is further compounded by the fact that pregnant women may not always discuss the information they find online with healthcare professionals, leading to gaps in understanding and potentially hindering effective treatment plans (22).

In conclusion, the knowledge, attitudes, and practices of pregnant women towards medications significantly influence their use. The objective of this study is to assess the knowledge, attitudes, and practices regarding medication use among pregnant women in Riyadh city, aiming to optimize healthcare for both pregnant women and their fetuses.

## Methodology

### Study design and population

This observational cross-sectional study utilized a self-administered questionnaire to gather data. The study population included all pregnant women residing in Riyadh City. Convenience and snowball sampling techniques were employed to recruit participants using WhatsApp and emails, and data were collected using an online questionnaire. The data collection period spanned one month, from March to April 2023. Inclusion criteria comprised all pregnant women living in Riyadh during the study period who consented to participate in the questionnaire.

### Data collection

The survey package, available in both Arabic and English, included details about the study and its purpose, informed consent, and the questionnaire itself. The questionnaire was adapted by Zaki and Albarraq in 2014 from two previously published surveys by Horne et al. and Nordeng et al. (11, 15, 24).

The questionnaire evaluated sociodemographic characteristics (Q1–5), awareness of risk (Q6–Q9), current medication use (Q10–Q13), and sources of drug information (Q14–Q18). Statements (G1–G7) were used to assess beliefs about medication using the Beliefs about Medicines Questionnaire (BMQ), while statements (S1–S8) evaluated attitudes and practices regarding medication use during pregnancy. The complete questionnaire is included in Appendix 1.

The questionnaire was designed in Arabic and English to accommodate both Arabic and non-Arabic-speaking participants. Its content validity was reviewed by an independent pharmacist and physician. Face validity was established through translation into Arabic by a certified translator, ensuring consistency between the English and Arabic versions. A pilot study involving 35 participants led to the rewording of two questions and the omission of one question based on participant feedback. A back-translation from Arabic to English confirmed that the text matched the original questionnaire.

### Ethical considerations

This study was approved by the Institutional Review Board of Alfaisal University, Riyadh, Saudi Arabia. The participants' identities remained anonymous throughout the study. Informed consent was obtained, and all participants were informed about the purpose of the study. Participants had the right to withdraw at any time without obligation to the study team. No incentives or rewards were given to the participants. There was no funding for the research.

### Statistical analysis

We estimated that there were 70,000 pregnant women in the Riyadh population. This was used to calculate the sample size assuming a 50:50 response to a Yes:No question (such as “Do you use prescribed drugs?” The 50:50 response odds is typically used since it provides the maximal variance for the binomial function, hence resulting in a conservative (i.e., high) sample size estimate. The resulting calculation gave a sample size of 383, which provided 5% margin of error (95% confidence interval). Thirty-five participants who took part in the pilot study were excluded from the final data analysis.

Data analysis was performed using Jamovi, version 2.4.0. Categorical data were presented as frequencies and percentages. Logistic regression was used to identify factors influencing the responses. Binomial and multinomial logistic regressions were conducted for questions and statements with sufficient response variability, defined as having at least two response categories differing by more than 10%. Disagreeing responses served as the reference level for all statements.

The analysis included the following predictors: age group, education level, number of pregnancies, nationality, and presence of chronic disease. The responses to the questions and statements were treated as dependent variables. Models were determined using stepwise regression, and all analyses were controlled for age, education level, and number of pregnancies. Collinearity for all models was checked and variance inflation factors <2. Outliers were checked for excess leverage. Only relationships significant at *p* < 0.01 were reported to mitigate against type I errors.

## Results

### Sociodemographic information

The study included 562 pregnant women residing in Riyadh City, Saudi Arabia. Among the participants, 310 (55.2%) were between 20 and 30 years old, and the majority (92.5%) were of Saudi nationality. Most participants, 524 (93.2%), had completed their university degrees. Regarding employment status, 289 (51.4%) were employed, and 88 (15.6%) worked in health-related fields. Among the participants, 275 (48.9%) were experiencing their first pregnancy (primigravida), 287 (51.1%) had experienced multiple pregnancies, and 80 participants (14.2%) had more than three pregnancies. Detailed sociodemographic information is presented in Table 1.

**Table 1:** Sociodemographic information of the participants (*n* = 562).

		*n*	%
Age (years)	Less than 20	2	0.4
	20–30	310	55.2
	31–40	223	39.7
	More than 41	27	4.8
Nationality	Saudi	520	92.5
	Non-Saudi	42	7.5
Education	Primary/secondary^a^	8	1.4
	High school	30	5.3
	University	524	93.2
Occupation	Housewife	273	48.6
	Healthcare related employee	88	15.7
	Employee (others)	201	35.8
Number of pregnancies	One	275	48.9
	2–3	207	36.8
	More than 3	80	14.2

^a^ Primary/secondary: any education level less than high school.

### Medication use

Among the participants, 10.5% (59 out of 562) reported having at least one chronic disease. The total number of chronic conditions was 66, including 15 different chronic disease categories. The most common chronic conditions were thyroid disorders, affecting 33.3% (22 out of 66 cases), followed by asthma at 10.6% (7 out of 66 cases), diabetes at 9.1% (6 out of 66 cases), and hypertension also at 9.1% (6 out of 66 cases), as detailed in Table 2.

**Table 2:** Frequency of chronic diseases (*n* = 66)^a^.

Disease	*n*	%
Thyroid disorders	22	33.3
Asthma	7	10.6
Diabetes	6	9.1
Hypertension	6	9.1
Inflammatory Bowel Disease	4	6.1
Autoimmune diseases	4	6.1
Polycystic ovary syndrome	3	4.5
Multiple sclerosis	3	4.5
Gastrointestinal tract/liver disorders	3	4.5
Musculoskeletal pain	2	3.0
Hematologic disorders	2	3.0
Hyperlipidemia	1	1.5
Migraines	1	1.5
Recurrent urinary tract infections	1	1.5
Others^b^	1	1.5

^a^ Because some women had more than one chronic disease, the total number in the table sums to 66.

^b^ Mitral valve regurgitation.

Additionally, 251 out of the 562 participants (44.7%) reported using at least one medication during their pregnancy (see Table 3). Besides vitamins and supplements, the most frequently used medications included hormones, aspirin/enoxaparin, levothyroxine, antimicrobials, antiemetics, antacids, analgesics/antipyretics, antidepressants, antihypertensives, antidiabetics, antihistamines, and inhalers, respectively.

**Table 3:** Medication used during pregnancy (*n* = 562).

		*n*	%
Using prescribed drugs	Yes	248	44.1
	No	314	55.9
Using non-prescribed drugs	Yes	66	11.7
	No	496	88.3

Regarding awareness of risk, the majority of participants, specifically 516 (91.8%), were aware of the most critical time during pregnancy (see Table 4). Additionally, about 60% of the participants knew which drugs should be avoided during pregnancy (as shown in Table 4). Notably, a significant majority of participants who used prescribed medications were aware of the indications for their medication, with 241 out of 248 (97.1%) demonstrating this level of awareness.

**Table 4:** Awareness of the risks (*n* = 562).

		*n*	%
Awareness of the critical time for drug use during pregnancy	First trimester	516	91.8
	Second trimester	23	4.1
	Third trimester	23	4.1
Awareness of the drugs should be avoided during pregnancy	Yes	340	60.5
	No	39	6.9
	Uncertain	183	32.6
Awareness of prescribed drug indication in case of use	Yes	241	97.1
	No	5	2
	Uncertain	2	0.8

### Source of information

**Figure 1:**
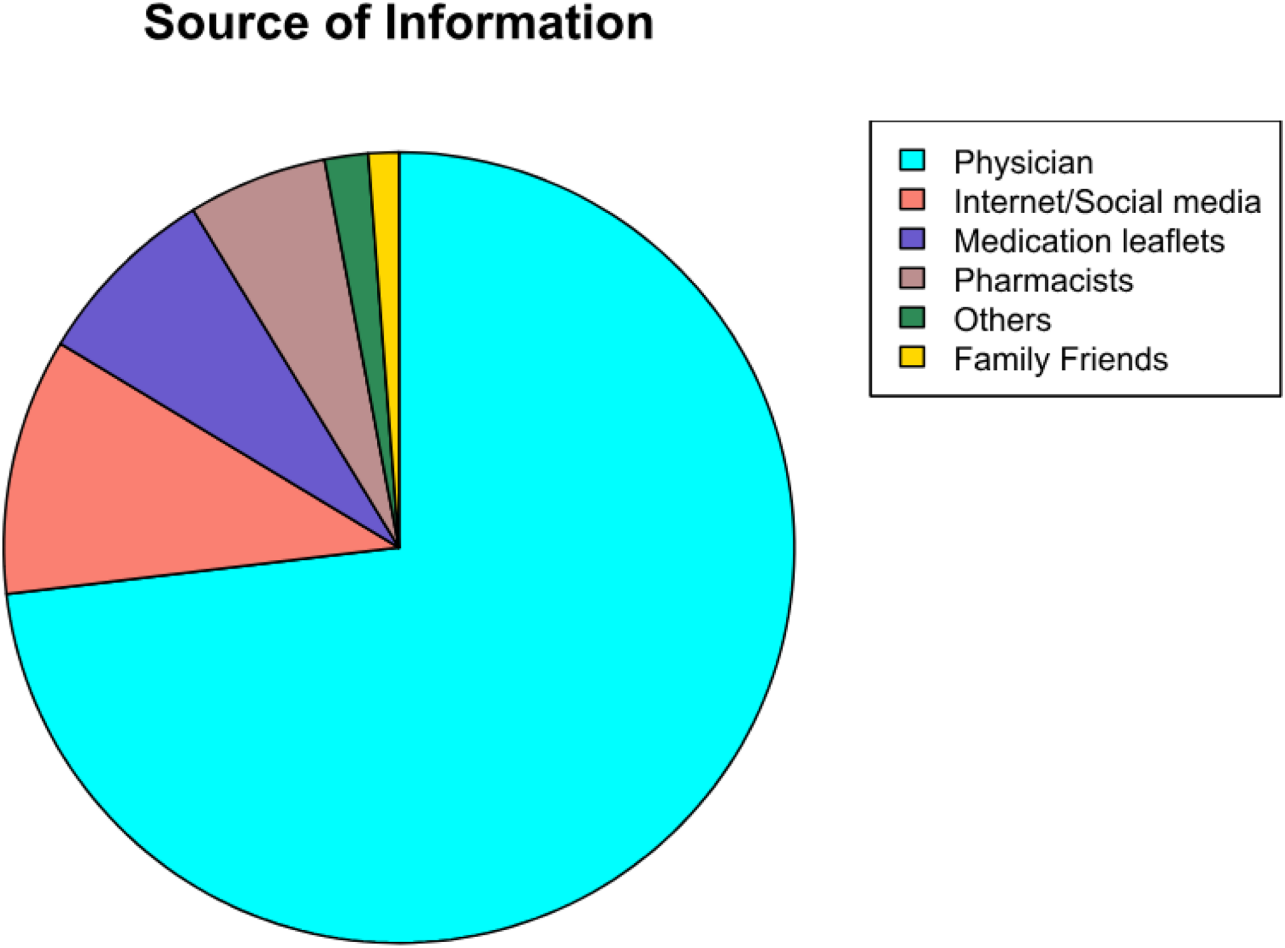
Source of drug information (*n* = 562).

As illustrated in Figure 1, 411 (73.1%) of the participants primarily rely on physicians for drug information, followed by the internet and social media 59 (10.5%), medication leaflets 43 (7.7%), pharmacists 32 (5.7%), family/friends 7 (1.2%), and others 10 (1.8%).

A substantial majority of the participants, numbering 539 (95.9%), reported regular visits to their physician during pregnancy. Among them, 232 (41.3%) indicated that they received sufficient information regarding their prescribed medications from their physician, whereas 193 (34.3%) reported receiving adequate information from the pharmacist at the time of medication dispensing. Furthermore, 423 (75.3%) of the participants stated that they read the medication leaflet when prescribed medication during their pregnancy.

### Beliefs about medications

Only 170 (30.2%) of the participants express the belief that doctors prescribe an excessive number of medicines, and 112 (19.9%) believe this applies specifically to pregnant women. Approximately 196 (35%) of the participants opined that doctors would prescribe fewer medications if they allocated more time to patients. Moreover, 359 (63.9%) perceive physicians to place excessive trust in medicines. A minority, comprising 38 (6.8%) of participants, perceive medications as addictive, while 5 (28%) regard all medicines as poisons; however, the majority disagree these notions. Additionally, 330 (58.7%) believe that the benefits of medicines outweigh their risks, while 69 (12.3%) belief that all medications are harmful to the fetus.

Regarding natural remedies, 157 (27.9%) perceive them to be safer than medicines in general. Moreover, 113 (20.1%) believe pregnant women can use them safely, although the majority disagree. Additionally, 153 (27.2%) of participants advocate for the use of natural remedies during pregnancy, while 351 (62.5%) believe that pregnant women should refrain from using them without a doctor's guidance.

Interestingly, 394 (70.1%) express a higher threshold for using medicines during pregnancy, and 401 (71.4%) opine that it is preferable to use medicines during pregnancy rather than leave illnesses untreated. Moreover, 364 (64.8%) of pregnant women believe that medicines have saved the lives of many unborn children. Further details are provided in Tables 5, 6.

**Table 5:** Beliefs about medication (general)- (*n* = 562).

Statement	Agree *n* (%)	Disagree *n* (%)	Uncertain *n* (%)
G1. Doctors prescribed too many medicines	170 (30.2)	264 (47.0)	128 (22.8)
G2. Most medicines are addictive	38 (6.8)	395 (70.3)	129 (23.0)
G3. Natural remedies are safer than medicines	157 (27.9)	240 (42.7)	165 (29.4)
G4. Medicines do more harm than good	94 (16.7)	330 (58.7)	138 (24.6)
G5. All medicines are poisons	28 (5.0)	449 (79.9)	85 (15.1)
G6. Doctors place too much trust in medicines	359 (63.9)	69 (12.3)	134 (23.8)
G7. If doctors had more time with patients, they would prescribe fewer medicines	196 (34.9)	180 (32.0)	186 (33.1)

**Table 6:** Beliefs about medication during pregnancy (specific)- (*n* = 562).

Statement	Agree *n* (%)	Disagree *n* (%)	Uncertain *n* (%)
S1. All the medicines can be harmful to the fetus	69 (12.3)	378 (67.3)	115 (20.5)
S2. I have a higher threshold for using medicines when I am pregnant than when I am not pregnant	394 (70.1)	103 (18.3)	65 (11.6)
S3. Thanks to treatment with medicines during pregnancy, the lives of many unborn children are saved each year	364 (64.8)	54 (9.6)	144 (25.6)
S4. It is better for the fetus that I use medicines and get well than to have untreated illness during pregnancy	401 (71.4)	57 (10.1)	104 (18.5)
S5. Doctors prescribe too many medicines to pregnant women	112 (19.9)	331 (58.9)	119 (21.2)
S6. Natural remedies can generally be used by pregnant women	113 (20.1)	287 (51.1)	162 (28.8)
S7. Pregnant women should preferable(y) use natural remedies during pregnancy	153 (27.2)	240 (42.7)	169 (30.1)
S8. Pregnant women should not use natural remedies without the advice of a doctor	351 (62.5)	165 (29.4)	46 (8.2)

### Predictors of medication use knowledge, attitude, and practice among pregnant women

**Figure 2:**
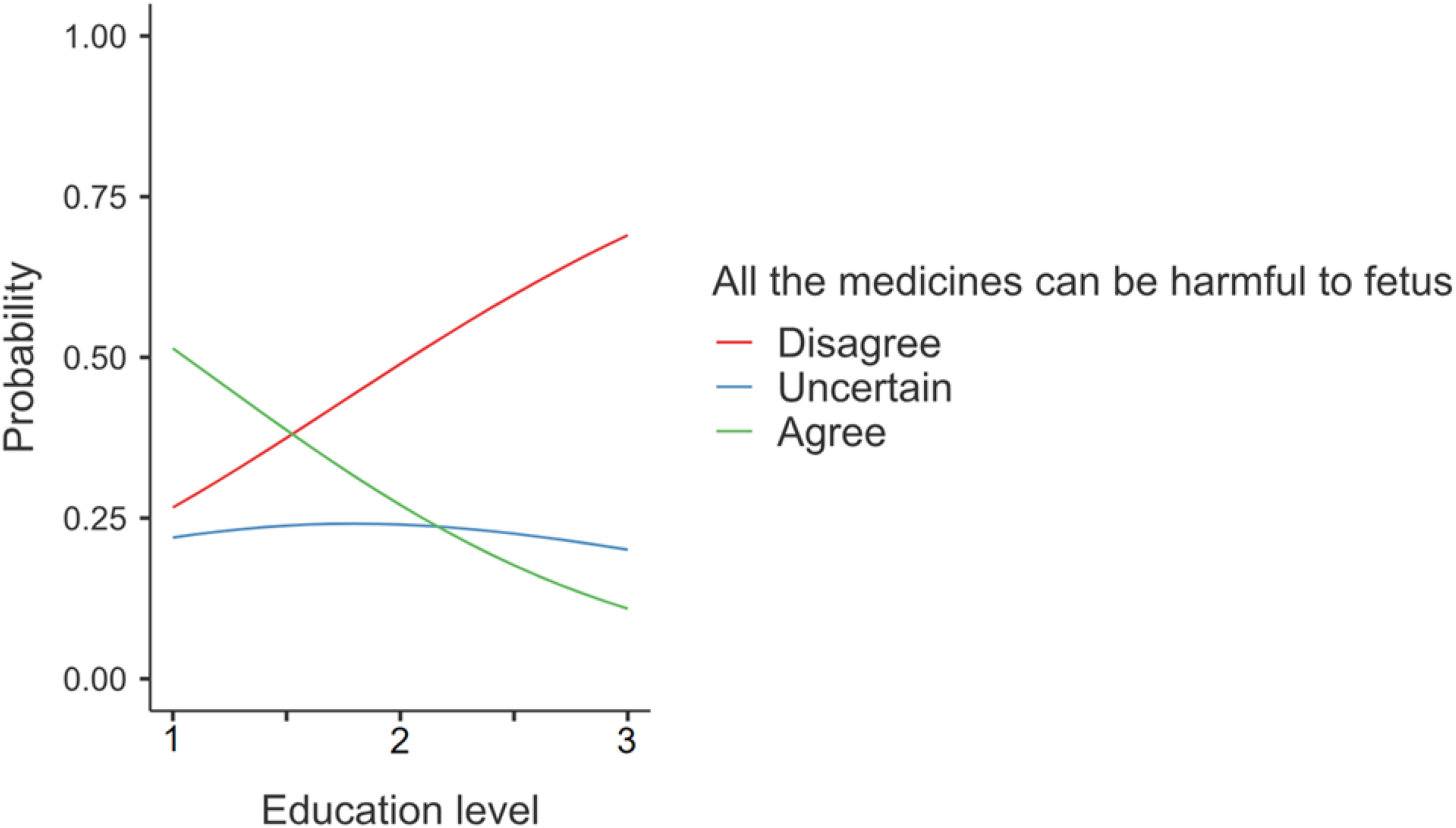
Responses on statement S1 “All the medicines can be harmful to the fetus”. The numbers given on the horizontal axis represent Education level: 1 = Primary/Secondary, 2 = High school, 3 = University.

Overall, the analysis revealed three notable associations between predictors and responses. Firstly, individuals with a university education were more likely to disagree with the statement that all medicines could harm the fetus (Statement S1), indicating a significant association between responses and the level of education (*z* = −3.79, *p* = 0.0001, OR = 0.3) (Figure 2).

**Figure 3:**
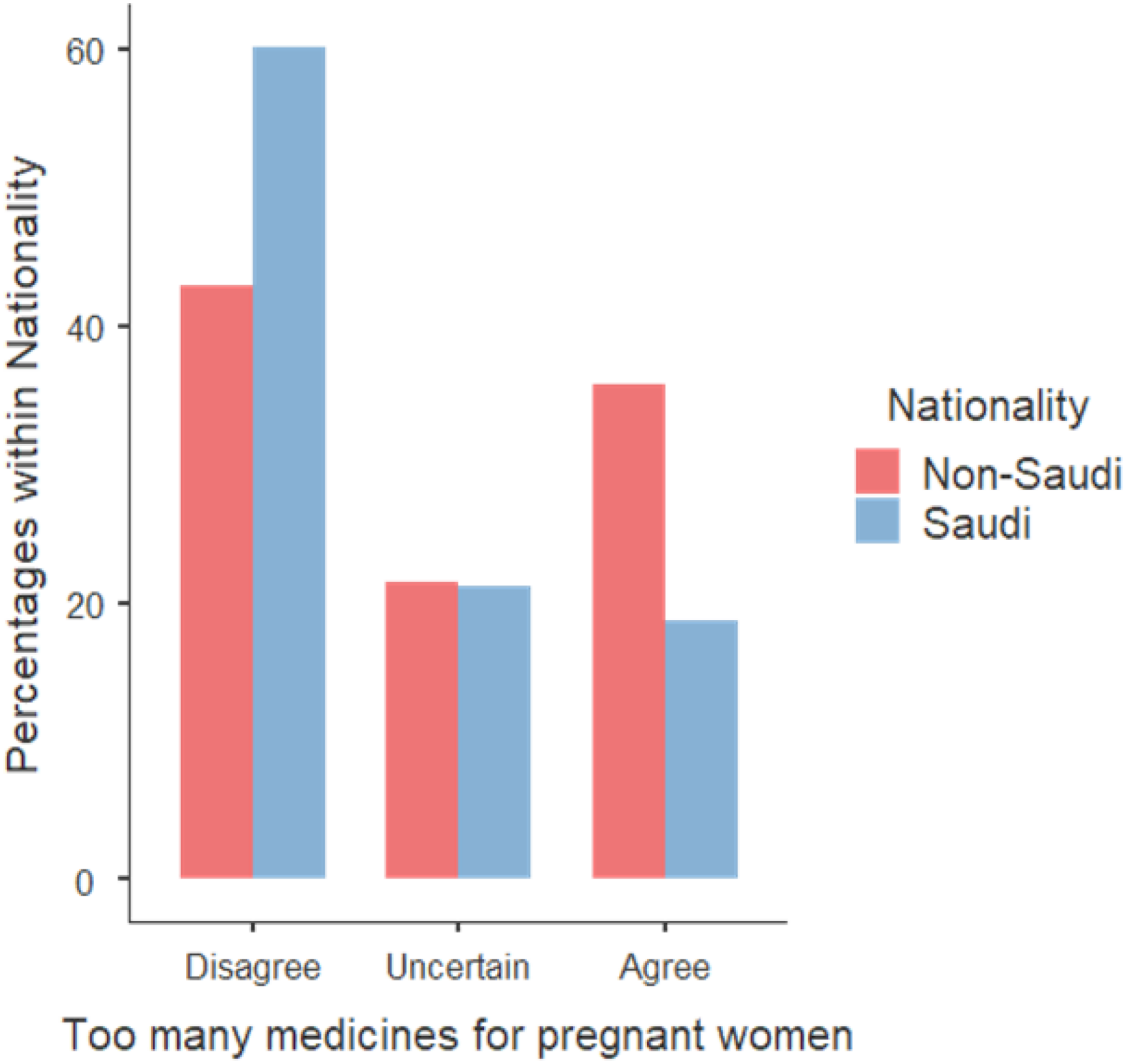
Responses on statement S5 “Doctors prescribe too many medicines to pregnant women”.

Secondly, Saudi women demonstrated a higher tendency to disagree with the notion that doctors prescribe too many medicines to pregnant women compared to non-Saudi women (Statement S5), highlighting a significant association (*z* = 2.63, *p* = 0.0086, OR = 2.66) (Figure 3).

**Figure 4:**
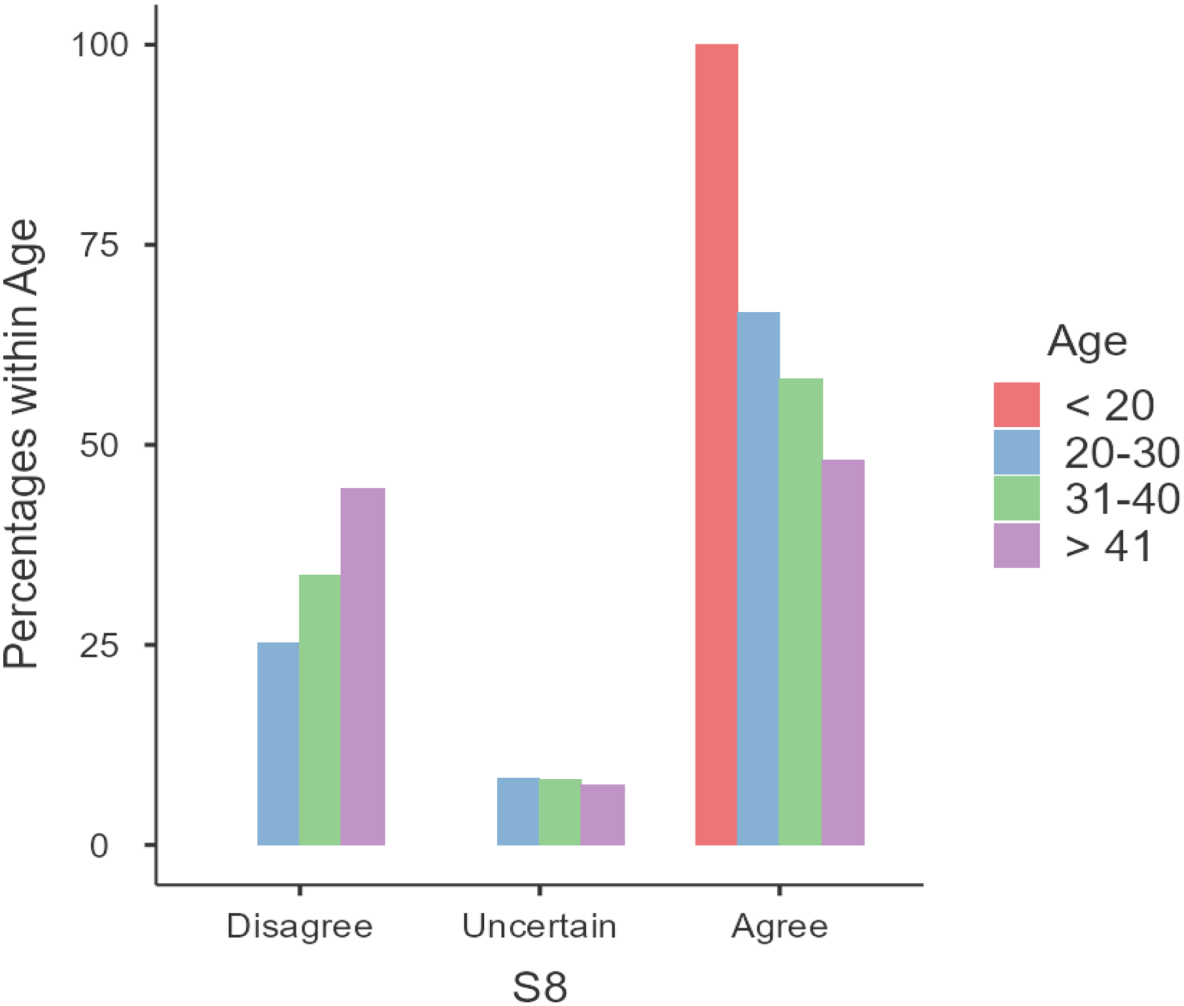
Responses to statement S8: “Pregnant women should not use natural remedies without the advice of a doctor”.

Lastly, age-related differences in responses were observed, particularly regarding the belief that pregnant women should not use natural remedies without a doctor's advice (Statement S8). Younger participants were more likely to agree with this statement (*z* = −3.31, *p* = 0.0009, OR = 0.54) (Figure 4). It is worth noting that there were only two pregnant women less than 20 years of age, both of whom agreed with the statement. Rerunning the statistical analyses excluding these two individuals yielded unchanged relationships.

Additionally, a sensitivity analysis was conducted, excluding healthcare-related employees (88 out of 562 participants), to mitigate potential bias due to medical knowledge among participants from the healthcare sector. Comparing response categories between the initial dataset and the one excluding healthcare-related employees revealed consistent results, with minimal, statistically insignificant differences of 1%–4% in some response categories.

## Discussion

In our study, 562 pregnant women residing in Riyadh City, Saudi Arabia, participated in an online questionnaire aimed at assessing their knowledge, attitude, and practice regarding medication use during pregnancy. The findings revealed a commendable level of knowledge and a positive attitude and practice towards medication use. However, a conservative attitude towards medication use during pregnancy was observed, consistent with prior reports by Zaki and Albarraq in the Saudi population. Our study did not find significant associations between sociodemographic variables and participants' responses, except for three specific statements. This finding is in line with the results of Obi and Anosike's study on the Nigerian population, where no associations were identified between sociodemographic variables and responses (25).

Demographic analysis of the participants revealed that the majority fell within the 20 to 30 age range, had a high level of education, and were of Saudi nationality. The prevalence of highly educated participants might reflect the broader literacy rates among women in Saudi Arabia, as reported by UNESCO with a 96.05% adult female literacy rate as of 2020 (26). However, it's worth noting that this high level of education may be inflated due to sampling techniques. Furthermore, nearly half of the participants were primigravida, and the majority reported good health, with only 10.5% indicating the presence of at least one chronic disease requiring chronic medication.

In terms of medication use during pregnancy, 44.7% of participants in our study reported utilizing at least one medication, a figure consistent with findings reported in Taif city by Zaki and Albarraq (approximately 40%), as well as documented rates in Ireland (46.8%) and Iceland (49%) (15, 27, 28). However, this percentage is lower compared to other studies, including those conducted in Italy (59.6%), France (89.9%), India (79.6%), Ethiopia (88.4%), Malaysia (81.4%), Canada (59%), Scotland (85.2%), and Norway (69.1%) (2, 29–35). Moreover, our percentage was higher than that reported in Australia (26.5%) (5). Additionally, 44.1% of participants reported using prescribed medications, while 11.7% acknowledged using medications without prescriptions, a prevalence notably higher in Italy, with a reported rate of 43.9%, as documented by Navaro et al. (35). Interestingly, a recently published study in Riyadh City by Alyami et al. reported that 76.66% of pregnant women used medications during pregnancy, with 86.36% of these being prescribed medications. However, this study was conducted at King Saud Medical City, a tertiary care hospital that likely includes more complicated pregnancy cases, which may explain the difference in results. According to the study, 47.7% of the participants had obstetric complications, and 19.51% had comorbid conditions (36).

Regarding awareness of risks, participants demonstrated a commendable level of knowledge, with the majority being aware of critical periods during pregnancy and drugs to avoid. Furthermore, a significant majority of participants using prescribed medications demonstrated awareness of their medication indications, indicating a remarkable level of awareness.

In terms of the source of drug information, our findings indicate that a majority of participants rely on physicians for medication information, followed by the internet/social media (10%), medication leaflets (8%), and pharmacists (6%). This reliance on physicians is notable, particularly given the potential risks associated with obtaining information from non-trusted sources on the internet and social media. This finding aligns with previous research by Zaki and Albarraq, where physicians were the primary source of medication information for 71.1% of participants, with the internet accounting for only 5% (37).

However, our findings contrast with other published data that emphasize the internet as a common resource for medication information among pregnant women. For instance, a study conducted in the UK found that 76% of participants had searched the internet for information on medication safety during pregnancy (38). Similarly, in Italy, 46.9% of participants identified the internet as their primary source of information on medication use during pregnancy (35).

Moreover, participants in our study demonstrated commendable practice by regularly attending physician visits during pregnancy. It is noteworthy that in Saudi Arabia, maternity services are provided for free through the public governmental sector or health insurance, potentially positively impacting this practice. However, a concerning aspect emerged as 58.7% of participants indicated a lack of adequate information about their prescribed medication from their physician, either not at all or not consistently. Additionally, 65.7% confirmed not receiving sufficient information from pharmacists during medication dispensing, or at least not consistently. This information deficiency may adversely impact their knowledge, attitude, and practice, aligning with the findings of Zaki and Albarraq (15). Also, it is aligned with the findings of Alyami et al. who reported that 40.77% of the participants did not receive any information about medication use during pregnancy (36). The insufficient information provided by physicians and pharmacists may be related to the knowledge gap identified in previous studies conducted in Saudi Arabia by Alshebly et al. and Alrabiah et al. (38, 39). Moreover, a systematic review examining patients' knowledge and sources of information regarding medication use during pregnancy found that the majority of women had information gaps concerning prescribed medications, regardless of the country. Five studies noted that patients believed the information provided by physicians was insufficient (40). This suggests a need for improved training and continuing education programs for healthcare professionals to ensure they have the knowledge to provide comprehensive and accurate patient information. Furthermore, in our study most participants mentioned that they routinely read the medication leaflet accompanying prescribed medication during pregnancy. This cautious approach is consistent with the findings of Zaki and Albarraq in Taif, Saudi Arabia (15), reflecting a hesitancy toward using medications during pregnancy, especially in the absence of comprehensive and trusted information provided by physicians and pharmacists during the prescribing and dispensing of medications.

Regarding beliefs about medication use during pregnancy, the overall survey responses reflect a positive outlook. This segment of the study utilized a BMQ-General and Specific questionnaire developed by Horne & Weinman (11) and Nordeng et al. (24), which have been employed in various studies across different communities. Notably, Zaki and Albarraq conducted a similar study in 2013 in Taif, Saudi Arabia, focusing on assessing medication use, knowledge, and beliefs among pregnant women. A detailed comparison of responses between our sample and theirs indicates both differences and agreements, as evidenced in Tables 7, 8. Additionally, for a more comprehensive comparison, we integrated data from a distinct population (Belgium), as presented in the study by Ceulemans et al. in 2019 (41). This additional layer of analysis provides a broader perspective on medication beliefs and practices among pregnant women across various populations.

**Table 7:** Beliefs about medication (general) comparison.

Statement		Agree (%)	Disagree (%)	Uncertain (%)
G1. Doctors prescribed too many medicines	Our study	30.2	47.0	22.8
	Zaki & Albarraq (15)	57.9	22.4	19.7
	Ceulemans et al. (41)	22.0	45.0	33.0
G2. Most medicines are addictive	Our study	6.8	70.3	23.0
	Zaki & Albarraq	26.3	57.9	15.8
	Ceulemans et al.	18.0	60.0	22.0
G3. Natural remedies are safer than medicines	Our study	27.9	42.7	29.4
	Zaki & Albarraq	44.7	28.9	26.3
	Ceulemans et al.	30.0	42.0	28.0
G4. Medicines do more harm than good	Our study	16.7	58.7	24.6
	Zaki & Albarraq	28.9	46.1	25.0
	Ceulemans et al.	4.0	77.0	19.0
G5. All medicines are poisons	Our study	5.0	79.9	15.1
	Zaki & Albarraq	26.3	48.7	25.0
	Ceulemans et al.	4.0	88.0	8.0
G6. Doctors place too much trust in medicines	Our study	63.9	12.3	23.8
	Zaki & Albarraq	67.1	15.8	17.1
	Ceulemans et al.	14.0	55.0	32.0
G7. If doctors had more time with patients, they would prescribe fewer medicines	Our study	34.9	32.0	33.1
	Zaki & Albarraq	52.6	30.3	17.1
	Ceulemans et al.	17.0	53.0	30.0

**Table 8:** Beliefs about medication during pregnancy (specific) comparison.

Statement		Agree (%)	Disagree (%)	Uncertain (%)
S1. All the medicines can be harmful to the fetus	Our study	12.3	67.3	20.5
	Zaki & Albarraq (15)	59.2	22.4	18.4
	Ceulemans et al. (41)	19.0	62.0	20.0
S2. I have a higher threshold for using medicines when I am pregnant than when I am not pregnant	Our study	70.1	18.3	11.6
	Zaki & Albarraq	88.2	6.6	4.3
	Ceulemans et al.	88.0	7.0	5.0
S3. Thanks to treatment with medicines during pregnancy, the lives of many unborn children are saved each year	Our study	64.8	9.6	25.6
	Zaki & Albarraq	46.1	32.9	21.1
	Ceulemans et al.	47.0	4.0	48.0
S4. It is better for the fetus that I use medicines and get well than to have untreated illness during pregnancy	Our study	71.4	10.1	18.5
	Zaki & Albarraq	44.7	31.6	23.7
	Ceulemans et al.	51.0	14.0	35.0
S5. Doctors prescribe too many medicines to pregnant women	Our study	19.9	58.9	21.2
	Zaki & Albarraq	19.7	61.8	18.4
	Ceulemans et al.	4.0	69.0	27.0
S6. Natural remedies can generally be used by pregnant women	Our study	20.1	51.1	28.8
	Zaki & Albarraq	21.1	59.2	19.7
	Ceulemans et al.	37.0	19.0	44.0
S7. Pregnant women should preferable(y) use natural remedies during pregnancy	Our study	27.2	42.7	30.1
	Zaki & Albarraq	25.0	48.7	26.3
	Ceulemans et al.	42.0	24.0	35.0
S8. Pregnant women should not use natural remedies without the advice of a doctor	Our study	62.5	29.4	8.2
	Zaki & Albarraq	75.0	11.8	13.2
	Ceulemans et al.	72.0	12.0	16.0

Approximately one-third of participants expressed the belief that doctors generally prescribe too many medicines, with around 20.0% specifically perceiving an excessive prescription rate for pregnant women. Notably, Saudi women exhibit a higher inclination to disagree with these statements compared to non-Saudi women. However, caution is warranted in interpreting this observation due to the limited number of non-Saudi participants. Additionally, roughly 35% of participants believe doctors would reduce medication prescriptions if they allocated more time to patients. Interestingly, 63.9% of participants believe physicians place excessive trust in medicines. These findings are consistent with Zaki and Albarraq's results, where over half of the participants expressed beliefs that doctors generally prescribe too many medicines and place excessive trust in them. Moreover, the responses to other statements were comparable (15). Interestingly, the study conducted in Belgium indicates lower agreement on the aforementioned statements, suggesting a higher level of trust and belief in the healthcare system. However, it is essential to acknowledge that both the Zaki and Albarraq, and Ceulemans et al. studies included participants from specific health facilities, potentially reflecting the influence of those facilities' practices on participants' beliefs.

Furthermore, a majority of participants in our study express positive beliefs, asserting that medications are neither addictive nor poisonous and that not all medications are harmful to the fetus. Additionally, over half of the participants believe that the benefits of medicines outweigh their risks in general. Notably, these responses align more closely with the findings of the Belgium study than the previous Saudi study, which reported a more skeptical attitude toward medications, particularly among participants with lower education levels. Significantly, our study identifies an association between responses reflecting beliefs about medication effects on the fetus and the level of education, suggesting a positive belief associated with a higher level of education. This influence of education on beliefs about medication use has been demonstrated in previous studies across different communities, including those in Saudi Arabia and Belgium (15, 41). It is noteworthy that, in terms of education level, our study population includes the highest number of participants with a high level of education compared to the other two studies.

The conservative attitude towards medication use among participants in the three distinct cohorts was comparable, with the majority expressing a higher threshold for using medicines during pregnancy compared to when not pregnant. However, the results of this study are reassuring, indicating that the majority of pregnant women do not believe that medications prescribed by physicians for medical conditions are poisonous and can lead to harm. Furthermore, most participants recognize the beneficial effects of prescribed medication for a medical condition, both for themselves and their fetus.

Regarding natural remedies, 20.1% of participants believe that pregnant women can use them, and 27.2% believe that pregnant women should use natural remedies during pregnancy. In Alyami et al, study only around 10% of the participants believed that most of the herbal medicine are safe (36). Our results align with those of the previous Saudi study, whereas the Belgian study indicates a higher preference for using natural remedies among pregnant women (15, 41). This difference may highlight the variations between the communities in general. However, the majority of participants across all discussed studies believe that pregnant women should not use natural remedies without a doctor's advice. Notably, age-dependent variations in responses were observed in our study, with the younger age group more likely to agree that pregnant women should not use natural remedies without the advice of a doctor.

Based on the study findings, implementing education and communication initiatives for healthcare professionals to enhance medication education activities, particularly in situations with limited information, will significantly impact public health. Furthermore, developing unified guidance with updated evidence-based information for healthcare professionals and pregnant women will be helpful to ensure the provision of trusted, consistent, and higher-quality evidence-based information.

### Strengths and limitations of the study

The study's strengths are multifaceted. First, it effectively identifies and addresses various dynamic aspects of women's attitudes and practices toward medication during pregnancy, providing a nuanced understanding of this critical issue. Second, the study ensures a more comprehensive and representative population by utilizing a large sample size and targeting pregnant women who receive diverse healthcare services from different providers. This approach enhances the generalizability of the findings. Last, using a questionnaire, previously utilized in other studies, facilitates the comparison of the findings across different populations and adds to the reliability of the study's outcomes.

However, the study is constrained by its observational design and sampling approach, including the dissemination channels for the questionnaire, which could have introduced biases, as well as the inability to quantify the response rate. The predominantly highly educated participant pool could potentially skew results positively, as observed in previous research. While employing anonymous self-reported surveys aids in mitigating social desirability bias, it remains susceptible to response bias. Furthermore, the accuracy of health status data and medication use history can be affected by self-reporting. Moreover, the lack of detailed information on pregnancy trimesters could limit the interpretation of results. The overrepresentation of primigravida participants and individuals without chronic health conditions might not fully reflect medication attitudes in more diverse populations. Lastly, the study's location in Riyadh city, the capital city of Saudi Arabia, may limit generalizability to regions with fewer healthcare resources.

## Conclusion

In conclusion, the present study offers valuable insights into the current landscape of knowledge, attitudes, and practices concerning medication use among pregnant women in the capital city of Saudi Arabia. While the overall findings suggest a positive outlook, there are discernible variations in responses indicating a degree of hesitancy and a cautious approach toward medication during pregnancy. Healthcare professionals need to acknowledge and account for this attitude when prescribing medications for pregnant women, as understanding the beliefs held by this demographic can significantly influence safety and outcomes.

Furthermore, the study highlights a concerning inadequacy in the education provided by healthcare professionals regarding medication use during pregnancy. This shortfall in information from frontline healthcare providers underscores an area for improvement aimed at enhancing the safety and effectiveness of medication use in pregnant women. Addressing this education gap holds the potential to positively impact the care and well-being of pregnant women.

## Data Availability

The original contributions presented in the study are included in the article/supplementary material, further inquiries can be directed to the corresponding author.

## Appendix 1

**Pregnant Women Knowledge, Attitude, and Practice of Medications Use in Riyadh City Study Survey**.

**Please fill in the required information and Tick the most accurate answer:**

**Sociodemographic information:**

1. Age:
  a. Less than 20 years
  b. b. 20–30
  c. c. 31–40
  d. More than 41
2. Nationality:
  a. Saudi
  b. Non-Saudi
3. Education level:
  a. Illiterate
  b. Primary/Secondary
  c. High School
  d. University
4. Occupation:
  a. Housewife
  b. Healthcare related employee
  c. Employee (others)
5. Number of pregnancies:
  a. 1
  b. 2–3
  c. More than 3

**Awareness about risks:**

6. Do you have any chronic diseases?
  a. Yes
  b. No
7. If yes, please specify which disease ….
8. What is the critical time for drug use during pregnancy?
  a. First trimester (1–3 months)
  b. Second trimester (4–6 months)
  c. Third trimester (7–9 months)
9. Are you aware of drugs that should be avoided during pregnancy?
  a. Yes
  b. No
  c. I'm not sure

**Medication use:**

10. Are using now prescribed drug/s during pregnancy?
  a. Yes
  b. No
11. If yes, specify the name of the drug/s ….
12. If yes, do you know why you are using this/these drug/s ….
  a. Yes
  b. No
  c. I’m not sure
13. Do you take any drugs without a prescription during pregnancy?
  a. Yes
  b. No

**Source of information:**

14. From where did you obtain drug information?
  a. Physician
  b. Pharmacist
  c. Medication leaflet
  d. Family/Friends
  e. Internet/social media
  f. Others
15. If you have a prescribed medication during pregnancy, do you read the medication leaflet?
  a. Yes
  b. No
  c. Sometimes
16. Do you visit your doctor regularly during pregnancy?
  a. Yes
  b. No
17. During medication prescribing, did the doctor give you adequate information about the prescribed drug/s?
  a. Yes
  b. No
  c. Sometimes
18. During medication dispensing, did the pharmacist give you adequate information about the prescribed drug/s?
  a. Yes
  b. No
  c. Sometimes

**Beliefs about medication in general:**

**Table:**

Statement	Agree	Disagree	Uncertain
G1. Doctors prescribed too many medicines			
G2. Most medicines are addictive			
G3. Natural remedies are safer than medicines			
G4. Medicines do more harm than good			
G5. All medicines are poisons			
G6. Doctors place too much trust in medicines			
G7. If doctors had more time with patients, they would prescribe fewer medicines			

**Beliefs about medication during pregnancy in specific:**

**Table:**

Statement	Agree	Disagree	Uncertain
S1. All the medicines can be harmful to the fetus			
S2. I have a higher threshold for using medicines when I’m pregnant than when I’m not pregnant			
S3. Thanks to treatment with medicines during pregnancy lives of many unborn children are saved each year			
S4. It is better for the fetus that I use medicines and get well than to have untreated illness during pregnancy			
S5. Doctors prescribe too many medicines to pregnant women			
S6. Natural remedies can generally be used by pregnant women			
S7. Pregnant women should preferable(y) use natural remedies during pregnancy			
S8. Pregnant women should not use natural remedies without the advice of a doctor			
